# The Mediation of Workplace Upward Networking on Affective Leadership and Employee Safety Voice Among Primary Nurses: A Cross-Sectional Study

**DOI:** 10.1155/jonm/5127212

**Published:** 2025-04-19

**Authors:** Danhong Qian, Xunyin Zhang, Lihui Yan, Yuqi Xu, Huijun Wang, Jinji Chen, Qi Zhou

**Affiliations:** ^1^Nursing Department, Hangzhou Linping District Integrated Traditional Chinese and Western Medicine Hospital, Hangzhou, Zhejiang, China; ^2^Nursing School, Lishui University, Hangzhou, Zhejiang, China

**Keywords:** affective leadership, employee safety voice, nurse, workplace upward networking

## Abstract

**Background:** Primary nurses hesitated to communicate directly with leaders about safety concerns due to apprehension about confrontation, fear of blame, and their undervalued opinions. Affective leadership's impact on voice behaviors was characterized by top–down impression management and an absence of upward management. This study aimed to validate the mediating role of workplace upward networking between affective leadership and employee voice behaviors.

**Methods:** This study was a cross-sectional survey, using purposive sampling to select 639 nurses from all primary hospitals in Hangzhou X district. Measurement tools included the demographic information questionnaire, affective leadership scale, employee safety voice scale, and workplace upward networking scale.

**Results:** The relationship between affective leadership and employee safety voice mediated by workplace upward networking was confirmed. Affective leadership affected workplace upward networking (*B* = 0.70, *p* < 0.0001), with an explained variation of 37%. Affective leadership and workplace upward networking affected employee safety voice (*B* = 0.45 and 0.21, *p* < 0.001), with an explained variation of 71%. Workplace upward networking's total, direct, and indirect effects were 0.65, 0.20, and 0.45, respectively.

**Conclusions:** The study offers a theoretical basis for enhancing the safety management framework in primary care. It highlights the importance of affective leadership, improving upward management systems and establishing a supportive safety culture to bolster nurse engagement and initiative in patient safety.

## 1. Background

Patient safety remains a serious issue worldwide, particularly in resource-constrained and understaffed primary care organizations, where safety incidents are more common [1, 2]. These problems jeopardize patient health and undermine public confidence in the healthcare system [3]. Research indicates that the absence of a safety culture is a fundamental source of patient safety issues. Hospital administrators handle patient safety issues mainly by adopting strict regulations and admonitions. However, establishing a safety culture requires active personnel involvement, with the employee safety voice being especially vital [4]. Employee safety voice is the active participation of employees in providing safety-related suggestions, feedback, or concerns, serving as the foundation for establishing a safe workplace [5]. As the “outpost” of patient safety, the safety voice of primary nurses can reveal potential hazards and promote safety improvement [6–9]. However, nurses are generally reluctant to speak up and exhibit low self-efficacy related to safety voice [10, 11]. They often face concerns while voicing their opinions, including the fear of negative attitudes and the lack of a supportive environment [12]. Therefore, knowing how to support and motivate primary nurses to voice safety is essential.

The current research is less focused on primary nurses' safety voices. Lee's meta-analysis showed that East Asian studies focused on South Korea, Hong Kong, and Taiwan, lacking reports from mainland China [11, 13]. Moreover, research in China has concentrated on large healthcare institutions, overlooking resource-limited primary healthcare institutions [5, 14]. Primary nurses face distinct challenges related to work stress and communication barriers. Conversely, primary managers lack advanced safety management experience and depend on their prior experience.

Multiple studies have shown that caring leaders may improve nurse safety voice [8]. Affective leadership (AL) is a style that emphasizes emotional engagement and support and is crucial for promoting employee safety [15]. AL involves people as free members of the multitude. Instead of remaining ignorant and passive to external factors, individuals actively enhance their ability to influence and be influenced [16, 17]. Management relies on strict and cold regulations, hindering nurses' active participation in security issues. The leader–member exchange (LMX) theory points out that a high-quality leader–member relationship increases interaction frequency and trust levels between employees and leaders, encouraging employees to proactively express safety-related suggestions or concerns [18]. AL motivates employees to reveal safety hazards and suggest improvements through active listening, emotional support, and resource security [5]. However, primary nurses' concerns about safety issues causing conflict, fear of being blamed, and feeling that their opinions may not be valued resulted in a lack of direct safety issues communication between nurses and leaders [5, 9, 19, 20]. As a result, AL tends to promote vocal behaviors by stimulating behavioral pathways in which employees initiate a close connection with their leaders.

The current research predominantly confirms a top–down mechanism between leadership and employee safety voices [21, 22], with less research on upward management [23]. Thus, the mechanism of upward management between AL and ESV is yet to be confirmed.

Workplace upward networking (WUN) is the behavior of an employee who obtains support resources or influences decisions by proactively establishing and maintaining contact with a leader [24]. WUN is characterized by an “upward pipeline” and an “upward influence.” The “upward pipeline” allows nurses to provide feedback on safety concerns quickly and safely by establishing formal and informal communication channels, lowering the psychological cost of voice [10–12]. The “upward influence” increases the presence and influence of nurses' opinions by strengthening a trusting relationship between nurses and their supervisors, making their vocal behaviors more efficacious [11, 25]. This dual effect improves communication between nurses and leaders and makes nurses' suggestions valued in decision-making, progressively establishing positive feedback and increasing safety voice's willingness and confidence. Therefore, it is essential to investigate the mediating role of WUN between AL and ESV.

In summary, this study aims to investigate the mechanism of action of AL through WUN to influence primary nurses' safety voice. This study can significantly mitigate safety risks, enhance communication efficiency, and provide a practical improvement path for daily safety management.

## 2. Methods

### 2.1. Design and Participants

This study was a cross-sectional survey using purposive sampling to select 639 nurses from all the Hangzhou Linping District primary hospitals. The inclusion criteria were as follows: (1) more than 1 year of clinical nursing experience, (2) more than six consecutive months of work in the department, and (3) voluntary participation in this study, and the exclusion criteria were advanced trainee and internship nurses.

### 2.2. Measurement

Demographic information includes gender, age, department, working experience, position, education, marital status, nursing attitudes, and appointment type.

AL was adopted from the AL scale compiled by Ching-Hsiung Qingxiong et al. in 2016 [15]. This version was contextualized and widely adopted in China to evaluate AL [26, 27]. The scale consists of 12 items divided into two dimensions: emotional care (6 entries) and emotional management (6 entries). The questionnaire uses a 5-point Likert scale with total scores ranging from 12 to 60. Higher scores reflect higher emotional leadership. The Wen et al. study reported a Cronbach's alpha of 0.939, which was 0.979 in the current study.

Upward management was adopted from the WUN scale compiled by Wang and Luan in 2024 [24]. This version was contextualized and widely adopted in China to evaluate upward management [28]. The scale consists of 8 items divided into two dimensions: task upward network (4 items) and personal upward network (4 items). The questionnaire uses a 5-point Likert scale, yielding scores between 8 and 40. Increased scores indicate enhanced upward management. A Cronbach's alpha of 0.92 was reported by Song Wang et al.'s study and was 0.915 in the current study.

Employee safety voice was adopted from the employee safety voice scale compiled by Sun et al. in 2022 [5]. This version was contextualized and widely adopted in China to evaluate employee safety voice [29, 30]. The scale consists of 11 items divided into two dimensions: promote safe voices (5 items) and prohibit safe voices (6 items). The questionnaire uses a 5-point Likert scale, yielding scores between 11 and 55. Increased scores indicate enhanced employee safety. A Cronbach's alpha of 0.90 was reported by Sun et al.'s study and was 0.97 in the current study.

### 2.3. Procedure

After obtaining approval from the institutional review board, the researchers visited the director of nursing, explained the purpose and procedures, and received permission. A trained research assistant contacted the nurses and distributed consent forms and questionnaires. All participants signed the informed consent form and completed the questionnaires.

### 2.4. Data Analysis

Data analysis for hypothesis testing was performed using Hayes' PROCESS and Hierarchical regression (SPSS 25.0 program with PROCESS Version 3.5). First, descriptive statistics were used to describe characteristics and instrument scores. Second, correlations between continuous variables were analyzed using Pearson correlation coefficients. Third, assumptions of linearity, normality, multicollinearity, and outliers were assessed to ensure the suitability of regression analysis. Fourth, the PROCESS macro (5000 and 10,000 bootstraps resamples) was applied using Model 4, with significant mediation effects identified when the 95% confidence interval excluded zero. Covariates were controlled, including age, work experience, position, and nursing attitudes, and study variables were standardized. Finally, hierarchical regression was performed by sequentially entering covariates (Step 1), AL (Step 2), and WUN (Step 3).

### 2.5. Ethical Considerations

Hangzhou Linping District's Integrated Traditional Chinese and Western Medicine Hospital (approval no. 2024-L-38) approved this study. The study's adherence to ethical principles will ultimately protect the participants' rights and privacy. This study followed the STROBE Guidelines.

## 3. Results

Of the 700 questionnaires distributed, 639 nurses provided data with a response rate of 91.2%. The majority of nurses were female (97%, *n* = 620), 26∼36 years old (41.7%, *n* = 216), bachelor's degree (88.6%, *n* = 566), 6–11 years working experience (27.1%, *n* = 173), senior nurse (45.4%, *n* = 290), liked nursing attitudes (53.1%, *n* = 339), held a Bianzhi (69.8%, *n* = 446), and married (80.3%, *n* = 513). Total mean ESV scores significantly differed by working experience, position, and nursing attitudes (Table 1).

The total mean score for AL was 4.20 ± 0.83, WUN was 3.64 ± 0.96, and employee safety voice was 4.22 ± 0.73 (Table 2).

The findings showed that there was a strong positive correlation between AL and WUN (*r* = 0.61, *p* < 0.001), WUN and ESV (*r* = 0.59, *p* < 0.001), and AL and ESV (*r* = 0.68, *p* < 0.001) (Table 3).

The Kolmogorov–Smirnov test confirms that the model's residuals follow a normal distribution (*p* > 0.05), which is also validated by the Q–Q plot. The variance inflation factor is 1.596 (< 2.0), with a tolerance of 0.627 (> 0.1), indicating no significant multicollinearity. Leverage values and Cook's distances are below 0.001 (< 0.02 for all cases), confirming no influential outliers. Hierarchical regression's *R*^2^ was 0.53 (*p* < 0.001).

The relationship between AL and ESV mediated by the WUN was confirmed. AL affected WUN (*B* = 0.70, *p* < 0.0001), with an explained variation of 37%. Hierarchical regression AL and WUN affected ESV (*B* = 0.45 and 0.21, *p* < 0.001), with an explained variation of 71%. WUN's total, direct, and indirect effects were 0.65, 0.20, and 0.45, respectively. All values were significant, indicating that WUN partially mediated between AL and ESV (Table 4). The mediation effect was reassessed using 10,000 bootstrap resamples, yielding nearly identical confidence intervals (WUN indirect effect: 95% CI: 0.16, 0.26), with a WUN effect size of 0.21.

## 4. Discussion

This study reveals first that WUN partially mediates the relationship between AL and ESV. Its findings can assist nursing managers in identifying how AL can successfully mitigate safety issues and enhance communication efficiency. In theoretical contributions, this study expands LMX theory by integrating WUN as a relational process that enhances the leader–employee dynamic, particularly in safety-critical healthcare. Second, this study challenges traditional unidirectional leadership frameworks and highlights the importance of WUN in promoting ESV among primary nurses. In practical implications, healthcare institutions should develop training programs that equip nursing managers with emotional support, trust-building, and active listening skills. Second, hospital managers should use anonymous reporting systems, scheduled leadership–nurse safety meetings, and open-door policies, ensuring that employees feel safe voicing concerns. Third, safety voice contributions should be incorporated as an evaluation metric in performance appraisals. Finally, a tutoring program and leadership development workshops should be provided to teach nurses how to establish effective communication with nursing supervisors.

This study revealed a high employee safety voice among nurses (mean score: 4.22 ± 0.73), surpassing that observed in workers [5]. This difference can be attributed to the healthcare sector's strong emphasis on patient safety, structured team communication, and professional training that fosters safety advocacy. In contrast, the construction industry's decentralized work arrangements and limited integration of safety voice into daily operations may account for lower engagement levels.

This study first verified a significant positive correlation (*r* = 0.61, *p* < 0.001) between AL and WUN in the workplace. The findings indicate that AL enhances employees' capacity to develop and sustain high-quality relationships. High LMX indicates that AL promotes employee initiative and interpersonal engagement by addressing emotional needs and fostering a trusting, safe work environment, thereby facilitating the development of upward management. The findings enhance the importance of AL in the social setting and deepen understanding of the mechanisms between leadership behavior and workplace relationship dynamics.

WUN and ESV had a strong positive correlation (*r* = 0.59, *p* < 0.001). This finding aligns with previous studies highlighting the relationship between task upward networks and ESV. However, these studies primarily emphasized task-related upward interactions while overlooking the role of personal upward networks [4, 31]. Notably, personal upward management also plays a significant role in influencing safety voice behaviors. Personal upward management significantly influences safety voice behaviors among primary nurses by fostering trust and psychological safety with their superiors. It enables nurses to communicate safety concerns openly, reducing fear of reprisal and empowering them to navigate hierarchical structures effectively in frontline healthcare settings. Strong interpersonal relationships with leaders enhance the receptivity of safety concerns raised by nurses, encouraging proactive communication and promoting a culture of critical patient care in primary healthcare environments.

AL and ESV had a strong positive correlation (*r* = 0.68, *p* < 0.001). This study is partially consistent with Krenz, Qasim, Tucker, and Conchie [13, 21, 32, 33]. All of these studies demonstrate the importance of leadership for ESV. AL fosters trust, psychological safety, and open communication among nurses, which is essential for identifying and addressing potential risks in the clinical environment. Moreover, AL fosters an organizational environment where nurses feel esteemed and supported, giving them a safe voice.

This study shows that WUN partially moderated the relationship between AL and ESV. This procedure is manifested in two stages: (1) AL significantly enhances the trust relationship between employees and leaders through caring, supportive, and authentic communication, which facilitates the establishment of WUN and (2) through the establishment of WUN, employees feel respected and valued in their work environments and are more willing to take the initiative to voice safety issues. AL cultivates a supportive environment for nurses, fostering a sense of value and trust and encouraging them to express their opinions regarding safety issues in a manner conducive to upward management. Second, nurses can propose patient safety recommendations and address work difficulties more confidently through this positive upward communication. Li's research confirmed the mediation function of impression management [34]. Through upward management, primary nurses could effectively communicate their insights, demonstrating personal value and team communication, which is crucial for promoting healthcare safety.

Resource-constrained primary healthcare settings should foster an ESV culture emphasizing AL and WUN. Accordingly, this study offers three recommendations for nursing management and decision-making. First, healthcare institutions should prioritize education and training to cultivate AL, focusing on emotional support, active listening, and trust-building. Second, formal upward management channels, such as anonymous reporting systems and regular safety briefings, should be institutionalized to mitigate the psychological barriers associated with ESV. Third, integrating ESV into performance evaluations can serve as an incentive for WUN.

This study has three limitations. First, the questionnaire has social desirability bias and subjective assessments. Future research may address this issue by incorporating longitudinal designs or triangulating data with other sources, such as leader-reported outcomes or observational methods. Second, results from primary hospitals in Hangzhou may restrict the findings to rural or tertiary healthcare institutions. It would be beneficial to discuss how the findings apply to other healthcare settings or regions and suggest areas for future research to explore these dynamics in diverse contexts. Third, the cross-sectional design precludes causal inference. Longitudinal studies should elucidate the temporal relationships among AL, WUN, and ESV.

## 5. Conclusions

This study's findings suggest that AL may motivate nurses to proactively report safety hazards and recommend improvements via upward management mechanisms. Meanwhile, WUN functioned as an essential bridging mechanism to improve the impact of nurses' safety voice decision-making. The study offers theoretical backing for optimizing the safety management model in primary care, highlighting the necessity of cultivating affective leaders, enhancing the upward management mechanism, and establishing a supportive safety culture in practice to boost nurses' engagement and initiative in patient safety.

## Figures and Tables

**Table 1:** Characteristics of the subjects and scores on employee safety voice (*N* = 639).

Variable	Categorization	Frequency	Percentage (%)	Mean	SD	*p*
Gender	Male	19	3	4.35	0.72	0.493
	Female	620	97	4.21	0.73	

Age (years)	< 26	62	9.7	4.21	0.68	0.071
	26∼< 36	285	44.6	4.25	0.75	
	36∼< 46	250	39.1	4.21	0.71	
	> 46	42	6.6	4.16	0.68	

Department	Internal medicine	109	17.1	4.22	0.77	0.825
	Surgery	90	14.1	4.50	0.55	
	Obstetrics	19	3	4.11	0.78	
	Emergency	38	5.9	4.31	0.66	
	ICU	41	6.4	4.31	0.74	
	Operating room	9	1.4	4.14	0.75	
	Outpatient	84	13.1	4.15	0.74	
	Other	249	39	4.14	0.74	

Working experience (years)	< 6	120	18.8	4.18	0.74	0.036
	6∼< 11	173	27.1	4.28	0.75	
	11∼< 16	168	26.3	4.23	0.72	
	16∼< 21	109	17.1	4.21	0.73	
	> 21	69	10.8	4.19	0.67	

Position	Nurse	78	12.2	4.24	0.75	0.018
	Senior nurse	290	45.4	4.22	0.75	
	Nurse practitioner in charge	239	37.4	4.21	0.71	
	Associate or chief nurse practitioner	32	5	4.29	0.58	

Education	Junior college	5	0.8	4.15	0.48	0.28
	College	59	9.2	4.10	0.81	
	Bachelor's degree	566	88.6	4.23	0.72	
	MD or PhD	9	1.4	4.58	0.55	

Marital status	Married	513	80.3	4.25	0.71	0.708
	Unmarried	107	16.7	4.10	0.80	
	Divorced	19	3	4.26	0.73	

Nursing attitudes	Likes	339	53.1	4.44	0.65	< 0.001
	Normal	294	46	3.98	0.73	
	Dislike	6	0.9	4.05	0.84	

Type of appointment	Bianzhi	446	69.8	4.23	0.73	0.157
	Contract	193	30.2	4.21	0.72	

**Table 2:** The mean scores of nurses on AL, WUN, and ESV (*n* = 639).

Variable	Mean	SD
Affective leadership (AL)	4.20	0.83
Workplace upward networking (WUN)	3.64	0.96
Employee safety voice (ESV)	4.22	0.73

**Table 3:** Variable relationships.

Correlation	AL	WUN	ESV
AL	1	0.61^∗∗^	0.68^∗∗^
WUN	0.61^∗∗^	1	0.59^∗∗^
ESV	0.68^∗∗^	0.59^∗∗^	1

*Note:* All correlations were significant at ^∗∗^*p* < 0.001.

**Table 4:** Mediation effect of AL between WUN and ESV.

	WUN				ESV			
	Coeff.	SE	*t*	*p*	Coeff.	SE	*t*	*p*
AL	0.70	0.04	23.82	*p* < 0.001	0.45	0.03	6.97	0.004
WUN					0.21	0.03	23.67	*p* < 0.001
Adjust *R*^2^	0.47				0.71			
*F*	567.66				789.83			

**AL ⟶ WUN ⟶ ESV**	**Effect**	**BootSE**	**Confidence interval**				**Efficiency ratio**	
			**Low bound**		**Upper bound**			

Indirect effect	0.45	0.04	0.39		0.52		0.70	
Direct effect	0.20	0.04	0.12		0.27		0.30	
Total effect	0.65	0.03	0.68		0.84			

*Note:* The working experience, position, and nursing attitudes were controlled as covariates.

## Data Availability

Research data are not shared.

## Nomenclature

IB: Innovation behavior
ESV: Employee safety voice
WUN: Workplace upward networking
